# Effective reduction of unnecessary biopsies through a deep-learning-assisted aggressive prostate cancer detector

**DOI:** 10.1038/s41598-025-99795-y

**Published:** 2025-04-30

**Authors:** Nuno M. Rodrigues, José Guilherme de Almeida, Ana Sofia Castro Verde, Ana Mascarenhas Gaivão, Carlos Bireiro, Inês Santiago, Joana Ip, Sara Belião, Celso Matos, Leonardo Vanneschi, Manolis Tsiknakis, Kostas Marias, Daniele Regge, Sara Silva, The ProCAncer-I Consortium, Manolis Tsiknakis, Kostas Marias, Stelios Sfakianakis, Varvara Kalokyri, Eleftherios Trivizakis, Grigorios Kalliatakis, Avtantil Dimitriadis, Dimitris Fotiadis, Nikolaos Tachos, Eugenia Mylona, Dimitris Zaridis, Charalampos Kalantzopoulos, Nikolaos Papanikolaou, José Guilherme de Almeida, Ana Castro Verde, Ana Carolina Rodrigues, Nuno Rodrigues, Miguel Chambel, Henkjan Huisman, Maarten de Rooij, Anindo Saha, Jasper J. Twilt, Jurgen Futterer, Luis Martí-Bonmatí, Leonor Cerdá-Alberich, Gloria Ribas, Silvia Navarro, Manuel Marfil, Emanuele Neri, Giacomo Aringhieri, Lorenzo Tumminello, Vincenzo Mendola, Deniz Akata, Mustafa Özmen, Ali Devrim Karaosmanoglu, Firat Atak, Musturay Karcaaltincaba, Joan C. Vilanova, Jurgita Usinskiene, Ruta Briediene, Audrius Untanas, Kristina Slidevska, Katsaros Vasilis, Georgiou Georgios, Dow-Mu Koh, Robby Emsley, Sharon Vit, Ana Ribeiro, Simon Doran, Tiaan Jacobs, Gracián García-Martí, Daniele Regge, Valentina Giannini, Simone Mazzetti, Giovanni Cappello, Giovanni Maimone, Valentina Napolitano, Sara Colantonio, Maria Antonietta Pascali, Eva Pachetti, Giulio del Corso, Danila Germanese, Andrea Berti, Gianluca Carloni, Jayashree Kalpathy-Cramer, Christopher Bridge, Joao Correia, Walter Hernandez, Zoi Giavri, Christos Pollalis, Dimitrios Agraniotis, Ana Jiménez Pastor, Jose Munuera Mora, Clara Saillant, Theresa Henne, Rodessa Marquez, Nickolas Papanikolaou

**Affiliations:** 1 Computational Clinical Imaging Group, Champalimaud Foundation, Lisbon, Portugal; 2 LASIGE, Faculty of Sciences, LASIGE, Faculdade de Ciências, Universidade de Lisboa, 1749--016 Lisboa, Portugal, Lisbon, Portugal; 3 Radiology Department, Champalimaud Clinical Center, Champalimaud Foundation, Lisbon, Portugal; 4 NOVA Information Management School (NOVA IMS), Universidade Nova de Lisboa, Campus de Campolide, 1070-312 Lisboa, Portugal; 5 Institute of Computer Science, Foundation for Research and Technology Hellas (FORTH), 700 13, Heraklion, Greece; 6 Department of Electrical and Computer Engineering, Hellenic Mediterranean University, 710 04, Heraklion, Greece; 7 Computational BioMedicine Laboratory (CBML), Institute of Computer Science, Foundation for Research and Technology-Hellas (FORTH), Heraklion, Greece; 8 Department of Radiology, Candiolo Cancer Institute, FPO-IRCCS, Strada Provinciale 142 Km 3.95, 10060 Candiolo, Italy; 9 Department of Surgical Sciences, University of Turin, 10124 Turin, Italy; 10 Department of Radiology, Royal Marsden Hospital, Sutton, UK; 11 Radboud, Nijmegen, Netherlands; 12 HULAFE-Biomedical Imaging Research Group, Medical Imaging Department, Instituto de Investigación Sanitaria La Fe, Hospital Universitari i Politecnic La Fe, Valencia, Spain; 13 Academic Radiology, Department of Translational Research, University of Pisa, Pisa, Italy; 14 Department of Radiology, Hacettepe, Ankara, Turkey; 15 Department of Radiology (IDI), Institute of Biomedical Research of Girona Dr. Josep Trueta (IDIBGI), Girona, Spain; 16 National Cancer Institute, Vilnius, Lithuania; 17 General Anti-Cancer and Oncological Hospital of Athens, Athens, Greece; 18 Radiology & AI Research Hub, Division of Radiotherapy and Imaging, The Institute of Cancer Research, The Royal Marsden NHS Foundation Trust, London, UK; 19 Quirónsalud Hospital/CIBERSAM, Valencia, Spain; 20 Institute of Information Science and Technologies of the National Reserch Council of Italy, Pisa, Italy; 21 Mass General Hospital, Boston, USA; 22 B3D, Birmingham, UK; 23 Advantis, Athens, Greece; 24 Quibim, S.L., Valencia, Spain; 25 Univie, Vienna, Austria

**Keywords:** Cancer imaging, Machine learning, Prostate cancer

## Abstract

Despite being one of the most prevalent cancers, prostate cancer (PCa) shows a significantly high survival rate, provided there is timely detection and treatment. Currently, several screening and diagnostic tests are required to be carried out in order to detect PCa. These tests are often invasive, requiring either a biopsy (Gleason score and ISUP) or blood tests (PSA). Computational methods have been shown to help this process, using multiparametric MRI (mpMRI) data to detect PCa, effectively providing value during the diagnosis and monitoring stages. While delineating lesions requires a high degree of experience and expertise from the radiologists, being subject to a high degree of inter-observer variability, often leading to inconsistent readings, these computational models can leverage the information from mpMRI to locate the lesions with a high degree of certainty. By considering as positive samples only those that have an ISUP$\ge$2 we can train aggressive index lesion detection models. The main advantage of this approach is that, by focusing only on aggressive disease, the output of such a model can also be seen as an indication for biopsy, effectively reducing unnecessary biopsy screenings. In this work, we utilize both the highly heterogeneous ProstateNet dataset, and the PI-CAI dataset, to develop accurate aggressive disease detection models.

## Introduction

Prostate cancer (PCa) is the most prevalent cancer in men and the second most prevalent across genders^1^. However, PCa is also characterized by a low mortality rate provided there is early detection, a key factor in ensuring positive treatment outcomes. While biopsies constitute an essential step in diagnosing and stratifying prostate cancer, false positives or incorrect risk assessments can lead to over-treatment. Together with treatment side effects, this may result in a loss of quality of life for the patients, making it imperative to carefully consider treatment choices^2^. The development of computer-aided diagnosis (CAD) models capable of providing “virtual biopsies” assisted by biparametric MRI (bpMRI) has the potential to reduce unnecessary biopsies and improve the risk assessment process. Indeed, the typical process for the recommendation of a biopsy consists of the analysis by an expert radiologist who will recommend a biopsy based on a positive (>2) or negative (<3) Prostate Imaging-Reporting and Data System (PI-RADS) score^3^, a process with a high rate of false positives^4^.

While the performance of automated systems is seldom as good as that of expert radiologists^5^, the latter commonly suffer from inter- and intra-expert variability^6,7^, which can be a limiting factor in deciding between performing or not performing a biopsy or even in choosing an appropriate treatment. Computational models have the benefit of producing consistent results provided the input data is identical, with the caveat that performance degradation is common when transferring models between scanner manufacturers^8^ or, in the case of prostate bpMRI, scanner manufacturers and the use of endorectal coil. However, some works have explored the benefits of using large multi-centric heterogeneous datasets to improve the robustness and performance of the models, effectively reducing the effects of domain-shift^9–11^.

Recent CAD models have shown potential in several clinical applications for PCa, from disease aggressiveness classification^12–14^ to lesion segmentation and detection^9,15–25^. However, these works seldom focus on unnecessary biopsy reduction, a clinical endpoint which has direct implications for patient care. Additionally, they tend to make use of single-centric datasets and rarely include a prospective validation of the developed models. Here, we make use of the publicly available PI-CAI^25,26^, as well as ProstateNet (https://prostatenet.eu), a large-scale multi-centric dataset of multiparametric prostate MRI to train aggressive lesion segmentation models. We show that using heterogeneous datasets leads to improved segmentation and lesion detection performance, and validate it using a hold-out test set. Through a simulated clinical feasibility analysis, we show how the combination of medical recommendations with our fully automatic models can lead to an effective reduction in the number of unnecessary biopsies with no significant reduction in Recall, effectively reducing the number of false positives. Finally, we validate all aspects of this approach using prospective data.

## Methods

### Data

In this study, two different datasets were used: PI-CAI^26^ and ProstateNet (also refered to as PNet). Each dataset is composed of a retrospective cohort, with ProstateNet also having a prospective cohort. The following are the descriptions of the datasets:

- *PI-CAI* is a collection of Biparametric MRI volumes that include T2W, DWI and ADC sequences. These samples were acquired by three Dutch clinical centers (Radboud University Medical Center (RUMC), Ziekenhuis Groep Twente (ZGT), University Medical Center Groningen (UMCG)), and one Norwegian center (Norwegian University of Science and Technology (NTNU)), plus the additional inclusion of 329 cases from the ProstateX dataset^27^. These clinical centers used only Siemens Healthineers or Philips Medical Systems-based 1.5Tor 3T MRI scanners with surface coils to acquire the images, following the Biparametric prostate MRI protocol^28^. As stated in the official document of the dataset^26^, ISUP values of 0 represent confirmed negatives or cases without the required 3-year follow-up. In total, 1009 biparametric sequences were used.
- *ProstateNet* (PNet) is a collection of Biparametric MRI volumes that include T2W, DWI and ADC sequences. These samples were acquired by 12 clinical partners of the Procancer-I project. These partners used Siemens (Aera, Skyra, Sola, Avanto, VIDA, Tim, Prisma, Veri, Symphony, Osirix), Philips (Ingenia, Achieva, Multiva) and GE scanners (Optima, Signa, DISCOVERY). Given that each centre has specific acquisition protocols, no single one was used across all mpMRI studies done. All labels were acquired manually, and for each sample, the label consists of the index lesion (mandatory) and additional lesions that the patient has (optional). ISUP values of 0 represent cases confirmed negative after 1 year of follow-up or non-confirmed cases. In total, 1484 biparametric sequences were used.

To maximize data variability, both datasets were combined into a global one, dubbed PNetCAI.  Table 1 shows the composition of the different retrospective datasets regarding scanner manufacturers and ISUP grades, while Table 2 does the same for the prospective cohort. The prospective cases were downloaded from the ProstateNet platform on February 26th 2024. From these numbers, $15\%$ of the samples were used as a hold-out test set, and the remaining were used for training, following a 5-fold cross-validation (CV) strategy.

**Table 1:** Stratification of samples of the retrospective data cohort. On the left, number of samples by scanner manufacturer and by ISUP score for the retrospective cohorts. On the right, number and proportion of samples on the training and test sets.

Scanners							
	Total	Siemens	Philips	GE	Toshiba	No data	
ProstateNet	1009	364	403	198	2	42	
PI-CAI	1484	1208	276	0	0	0	
ISUPs							
	0	1	2	3	4	5	
ProstateNet	519	228	141	69	20	31	
PI-CAI	847	228	223	98	39	49	
ISUPs	0	1	2	3	4	5	
	Train						
ProstateNet	442 0.52	192 0.22	118 0.14	61 0.07	15 0.02	29 0.03	# samples proportion
PI-CAI	719 0.57	194 0.15	189 0.15	84 0.07	33 0.03	42 0.03	
	Test						
ProstateNet	77 0.51	36 0.24	23 0.15	9 0.06	5 0.03	2 0.01	
PI-CAI	128 0.57	34 0.15	34 0.15	14 0.06	5 0.03	7 0.03	

**Fig. 1:**
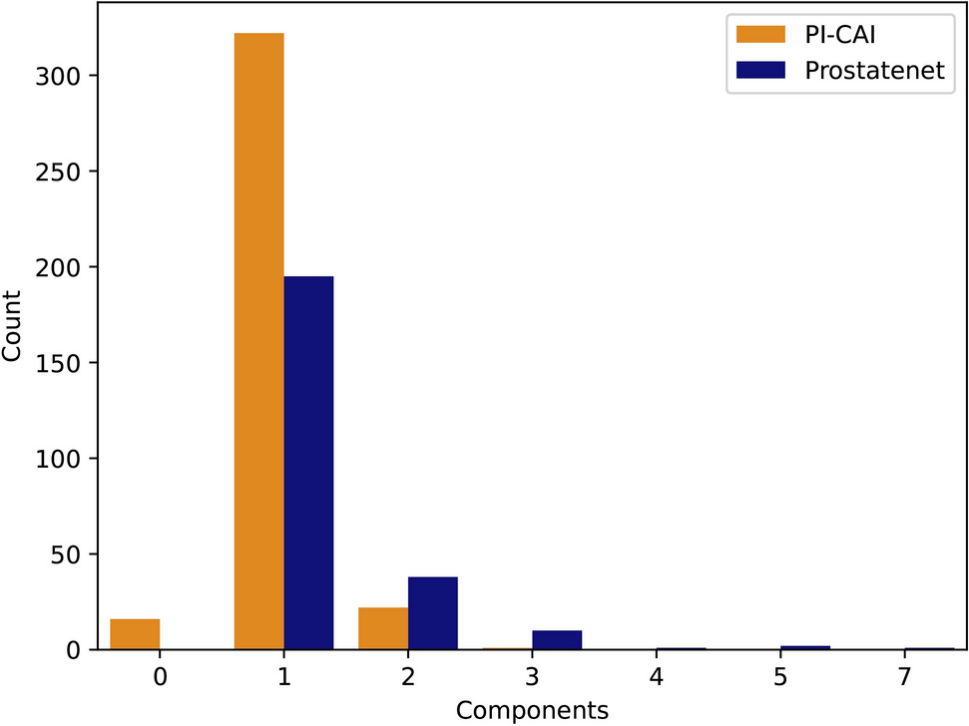
Connected component analysis. Connected components analysis for both aggressive (ISUP $\ge$ 2) label masks of the ProstateNet and PI-CAI datasets.

A connected component analysis was conducted on the training labels of both datasets ( Fig. 1), revealing that 16 samples from the PI-CAI datasets that were labelled as aggressive (ISUP $\ge$ 2) were empty. This was cross-checked with the files present in their repository. A comparison between the size of the lesions on both datasets and their effect on the Dice score is presented in the “Results” section (3).

**Table 2:** Stratification of samples by scanner manufacturer and ISUP score for the prospective cohort of ProstateNet.

Scanners						
Total	Siemens	Philips	GE	Toshiba	No data	
73	34	14	25	0	0	
ISUPs						
Total	0	1	2	3	4	5
73	36	16	7	9	5	0

### Biparametric data processing

In order to use all mpMRI sequences as a single volume, both DWI and ADC sequences were resampled to the same space and size of the T2W sequences. Both T2W and DWI images were normalized using Z-scoring normalization, while ADC images were normalized by clipping the intensity values to the 0.5 and 99.5 percentiles, followed by subtracting the mean and dividing by the standard deviation.

### Deep learning model specification

All 3D deep-learning (DL) detection models that were trained were full resolution nnUNet models (nnUNet)^29^ that use deep supervision^30^. The networks are implemented in Pytorch^31^ and were trained for 1000 epochs (250 mini-batches per epoch). To train the nnUNet models, we used the provided 3D full resolution architecture. This framework uses stochastic gradient descent with Nesterov momentum $(\mu =0.99)$, a maximum initial learning rate of 0.001, and polynomial^32^ learning rate policy which reduces the learning rate by a factor of $(1 - epoch/epoch_{max})^{0.9}$ in each epoch. Initial tests showed that the default learning rate of the nnUNet (0.01) was too high, resulting in underfitting on some of the folds, the reason why we decided to use a lower, more common, value. The loss function was a simple average of Dice and cross-entropy losses and the batch size was 2 sequences per iteration. The nnUNet applies automatic preprocessing based on the dataset fingerprint, and therefore the models for each dataset worked on data with slightly different spatial structures:

- ProstateNet: spacing = $0.5\times 0.5\times 3.0$mm; crop size = $256\times 256\times 30$ voxels
- PI-CAI: spacing = $0.4\times 0.4\times 3.0$mm; crop size = $384\times 384\times 21$ voxels
- PNetCAI: spacing = $0.5\times 0.5\times 3.0$mm; crop size = $384\times 384\times 23$ voxels

**Fig. 2:**
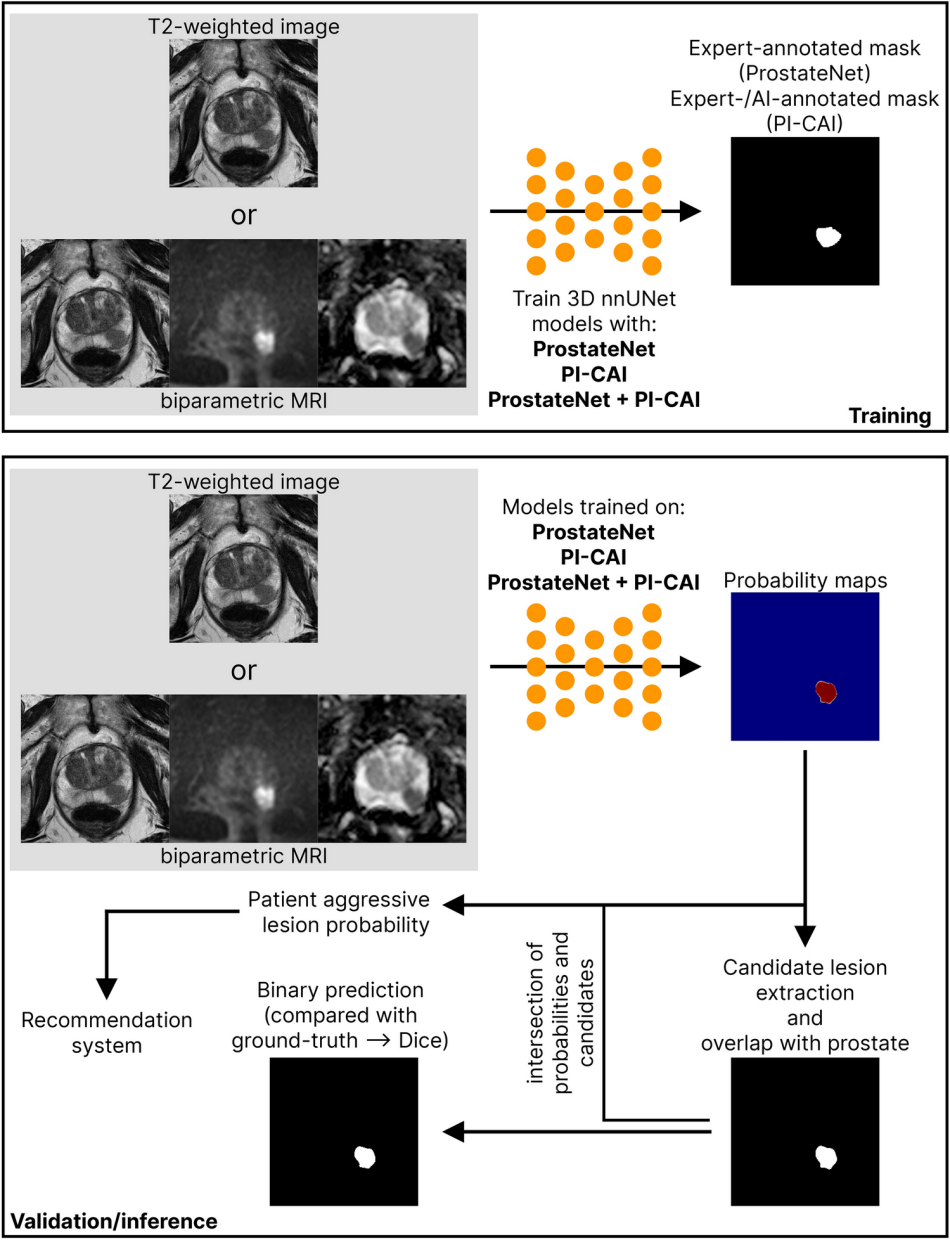
Visualization of the training and validation/inference protocol for the models described in this work. Training was performed using either T2-weighted or biparametric MRI studies belonging to either ProstateNet (PNet), PI-CAI or ProstateNet + PI-CAI (PNetCAI) to detect lesions annotated by radiologists. The validation/inference protocol consists in detecting lesions, extracting the most relevant lesion candidates^37^ and considering only lesions with an overlap of at least 10% with the whole prostate gland as inferred by a deep-learning model for prostate segmentation^11^. The patient aggressive lesion probability is then used in a recommendation system, while the binary/probabilistic prediction is used for visualization.

Based on recent work^11,33^, no transformer-based models (ViT) were evaluated, as they were shown to perform significantly worse than nnUNet models. This is further justified by the original ViT paper, which states the need for very large datasets (over 1 million images) to train a ViT model from scratch^34^.

#### Network calibration

Previous work^35^ and prior experiments conducted by us for whole gland segmentation have shown that calibrating segmentation models significantly improves their performance. Given this, we decided to use the findings from Murugresan et al.^35^ and change the nnUNet loss function to include both label-smoothing^36^ and margin loss. We applied an $\alpha$ smoothing factor of 0.2 and a margin of 10 to the loss function.

#### Technical specifications

To train the models for this project, we used a machine with the following specifications: 2$\times$ NVIDIA RTX A6000 GPUs, AMD Ryzen Threadripper 3990X 64-Core Processor, and 64GB DDR4 RAM with 2200MHz clock speed. Each fold of each model took approximately 13h to finish.

### Model evaluation

During the 5-fold CV, each model was evaluated based on its Dice Score (DS) and Recall when comparing the predicted output mask to that of the ground truth. When evaluating the performance on both the retrospective hold-out test and the prospective cohort, the same metrics were not computed on the vanilla output of the model, but on the candidate lesions obtained by following the subsequent methodology:

1. Taking the probability maps that the model outputs, a threshold of 10% was defined, clipping all voxels with a probability lower than 10%, generating a soft blob;
2. Taking those soft blobs, we employed the heuristics proposed by Bosma^37^ and assigned all lesion candidates to their respective ground truth through a linear sum assignment algorithm;
3. All candidates that had a confidence above 10% (the confidence is the maximum probability within the candidate) were kept and turned into hard blobs (binary segmentation masks). All other candidates (i.e. candidates with a confidence below 10%) were excluded and not analyzed any further. This threshold was selected as it reflects what has been used previously in the literature for prostate lesion candidate selection^37^;
4. Lastly, all hard blobs that had an intersection with the prostate gland of less than 10% (meaning they should be almost entirely outside the prostate, while still accounting for extracapsular extension) were classified as negative. The segmentations for the prostate gland were obtained using the whole gland segmentation model dubbed ProstateAll from Rodrigues et al.^11^;
5. In order to perform a more rigorous assessment, only hard blobs with at least 10% intersection with the original lesion masks were considered positive, regardless of having located any other lesion present in the same sample. This assessment, despite lowering some of the scores as opposed to simply locating any lesion, provides a more realistic clinical application scenario.

Each model was tested in all available retrospective hold-out sets and on the prospective cohort. The training/testing setup is summarized in Figure 2.

**Fig. 3:**
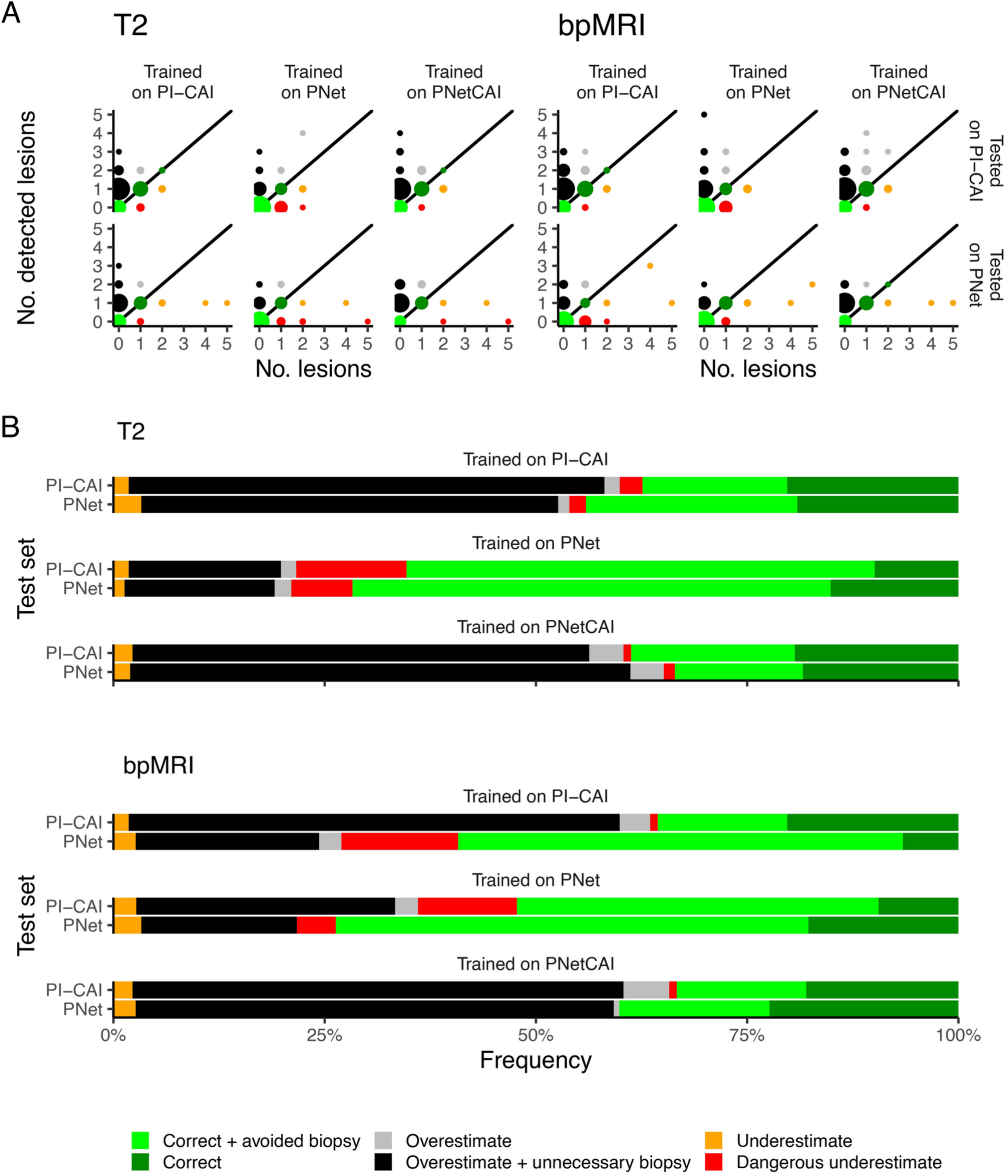
Distribution of CAD recommendations, stratified by training and testing dataset. (**A**) Distribution of annotated (no. of lesions in x-axes) and detected (no. of detected lesions in y-axes) lesions. (**B**) Relative frequencies of different predictions from the CAD system. For both (**A**,**B**) the colors correspond to a classification relating to whether or not this recommendation would lead to a change in the diagnostic algorithm proposed to the patient.

Additionally, we also calculated the Hausdorff Distance (HD), Average Symmetric Surface distance (ASSD), and Relative Absolute Volume Difference (RAVD) during quality assessment of the model, as these metrics provide a quantitative measure of the spatial accuracy by considering the shape and volume of the segmented regions^38^ (both distance metrics were calculated using MedPy^39^). The evaluations and details of each metric are available in the Supplementary Methods (A.1).

## Results

### Model performance is affected by train-test similarity

As previously mentioned in “Model evaluation”, we follow a two-step process in order to select the most appropriate lesion candidates: lesion candidates are selected similarly to what has been described in^37^, followed by a lesion filtering process that keeps only lesions with a 10% overlap with the whole prostate gland. Table 3 presents the cross-validation results of all developed models. Given that the models were trained as regular index lesion segmentation models, the resulting low Dice scores are a likely consequence of the heterogeneous nature of lesion annotation for the datasets used during training. We also note that bpMRI models outperform T2W models; this is expected, as both DWI and ADC sequences provide information in the form of hyper- and hypo-intense areas, which is much more relevant for lesion localization when compared to T2W sequences. The Recall also shows that bpMRI models, in particular the PI-CAI and PNetCAI models, can detect almost all lesions, achieving a maximum Recall score of 0.9 (90%), while their respective T2W counterparts can only locate approximately 65% of the lesions.

**Table 3:**
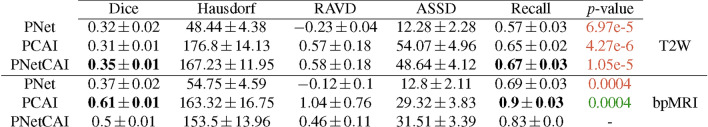
CV results. For each dataset, the average Dice, Hausdorf, RAVD, ASSD and Recall performance, along with their respective standard deviations, are presented. The highest recall value per sequence combination is highlighted in bold for easier comparison. p-values for the T-test significance comparing the Dice score between bpMRI PNetCAI results and each other model are also shown, with significant differences (*p*-value $< 0.01$ ) marked as green or red if the bpMRI PNetCAI results are better or worse, respectively.

The similarity between training and testing data (i.e., training and testing models on training and hold-out datasets constructed from the same dataset) can also be an important factor affecting performance. While T2W models trained on PNet data perform well only on data from PNet ($\textrm{Dice} = 0.34$ and $\textrm{Dice} = 0.13$ for T2W PNet models tested on PNet and PI-CAI, respectively), PI-CAI are more consistent ($\textrm{Dice} = 0.34$ and $\textrm{Dice} = 0.30$ for T2W PI-CAI models tested on PNet and PI-CAI, respectively; Tables 4, 5), an effect which is also consistent for Recall. However, using bpMRI leads to considerably worse performance in terms of both Dice and Recall for PI-CAI models tested on PNet data (Tables 4, 5); indeed, for bpMRI models, which outperform T2W models, performance is only consistently good for PNetCAI models. In other words, models perform consistently better only when there is some similarity between training and testing data.

**Fig. 4:**
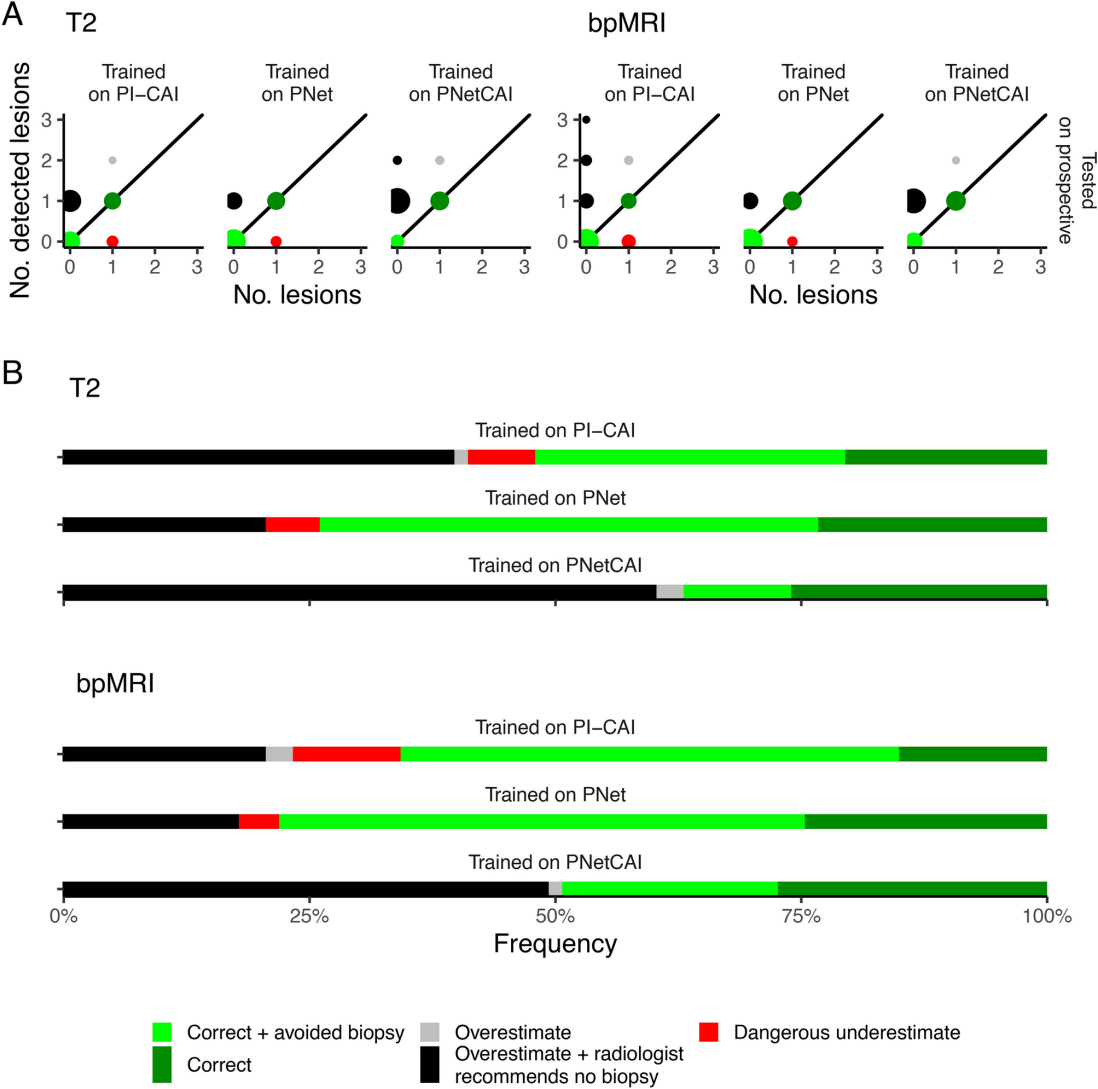
Distribution of CAD recommendations, stratified by training for the prospective dataset. (**A**) Distribution of annotated (no. of lesions in x-axes) and detected (no. of detected lesions in y-axes) lesions. (**B**) Relative frequencies of different predictions from the CAD system. For both (**A**,**B**) the colors correspond to a classification relating to whether or not this recommendation would lead to a change in the diagnostic algorithm proposed to the patient.

This can be further observed in Table 6, where the bpMRI PNetCAI excels over the bpMRI PNet model on its hold-out test set, while differing only in 2 lesions from the bpMRI PI-CAI model on its test set. Furthermore, after a manual analysis of these missed cases, we discovered that both where from out-of-distribution samples with very large fields of view.

**Table 4:** Hold-out test set results. For each pairwise evaluation, the average Dice, Recall and Precision performances are presented. The best Recall result for each dataset per sequence combination is highlighted in bold for easier comparison.

Trained on		PNet	PI-CAI	PNetCAI	
		$0.34 \pm 0.05$	$0.13 \pm 0.03$	$0.21 \pm 0.03$	Dice
		0.62	0.34	0.45	Recall
	PNet	0.47	0.33	0.4	Precision
		$0.34 \pm 0.05$	$0.3 \pm 0.03$	$0.32 \pm 0.03$	
		0.59	0.66	0.63	
	PI-CAI	0.24	0.24	0.24	
		$0.43 \pm 0.05$	$0.29 \pm 0.04$	$0.35 \pm 0.03$	
		0.74	0.64	0.68	
T2W	PNetCAI	0.25	0.24	0.24	
		$0.38 \pm 0.05$	$0.12 \pm 0.02$	$0.22 \pm 0.03$	
		0.72	0.34	0.49	
	PNet	0.5	0.23	0.33	
		$0.1 \pm 0.03$	$0.6 \pm 0.04$	$0.41 \pm 0.04$	
		0.33	**0.85**	0.64	
	PI-CAI	0.28	0.28	0.28	
		$0.41 \pm 0.04$	$0.53 \pm 0.04$	$0.49 \pm 0.03$	
		**0.79**	0.83	**0.82**	
bpMRI	PNetCAI	0.28	0.28	0.28

**Table 5:**
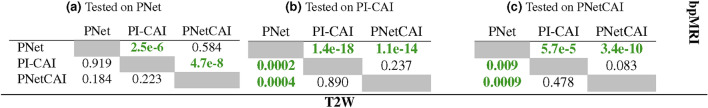
T-test *p*-values for the pairwise comparison of the Dice scores presented in Table 4. Significant differences (*p*-value $< 0.01$ ) marked as green.

**Table 6:** Hold-out test set results stratified by the ISUP grade of the lesions. For each pairwise evaluation, the number of predicted lesions is compared to the total number of lesions. The best-performing model (most successful detections) for each dataset per sequence combination is highlighted in bold.

Trained on	Tested on								
	PNet					PI-CAI			
T2W	PNet	14	5	3	2	12	3	1	4
	PI-CAI	11	6	5	2	21	7	5	6
	PNetCAI	15	7	5	2	22	3	3	6
bpMRI	PNet	13	8	5	2	11	4	2	3
	PI-CAI	7	5	1	0	**27**	**13**	**5**	**6**
	PNetCAI	**15**	**9**	**5**	**2**	27	11	5	6
# lesions		23	9	5	2	33	14	6	7
ISUP		2	3	4	5				

### Trade-off between avoiding biopsies and dangerous underestimates

To understand whether the best performing model—trained on bpMRI PNetCAI data—could be used as a CAD system for the effective reduction of biopsies (i.e. correctly predicting when an individual has no aggressive lesions), we first determined how many lesions were present in each case and calculated the number of detected lesions for all models. We then performed a simple experiment assigning lesions to one of six categories:

- Correct + avoided biopsy: if no lesions were present and the model correctly estimated this (i.e. recommended avoiding an unnecessary biopsy);
- Correct: if one or more lesions were present and the model correctly estimated the number of lesions
- Overestimate: if one or more lesions were present and the model overestimated the number of lesions
- Overestimate + unnecessary biopsy: if no lesions were present and the model overestimated the number of lesions (i.e. recommended an unnecessary biopsy)
- Underestimate: if two or more lesions were present and the model estimated a number of lesions between one and excluding the correct number of lesions
- Dangerous underestimate: if two or more lesions were present and the model detected no lesions (i.e. recommended avoiding a necessary biopsy)

This categorization system leads to a consistent trade-off between overestimating the number of lesions while recommending an unnecessary biopsy and avoiding unnecessary biopsies (Fig. 3); in other words, these systems could have the potential of reducing the number of biopsies but this set up has to be carefully considered as it could also result in avoiding biopsies for patients who would require them. A concerning aspect of this analysis is that only in one instance—PNetCAI models tested on PNet data—does it fulfill the task of reducing the number of biopsies without missing any relevant predictions (Table 7).

**Fig. 5:**
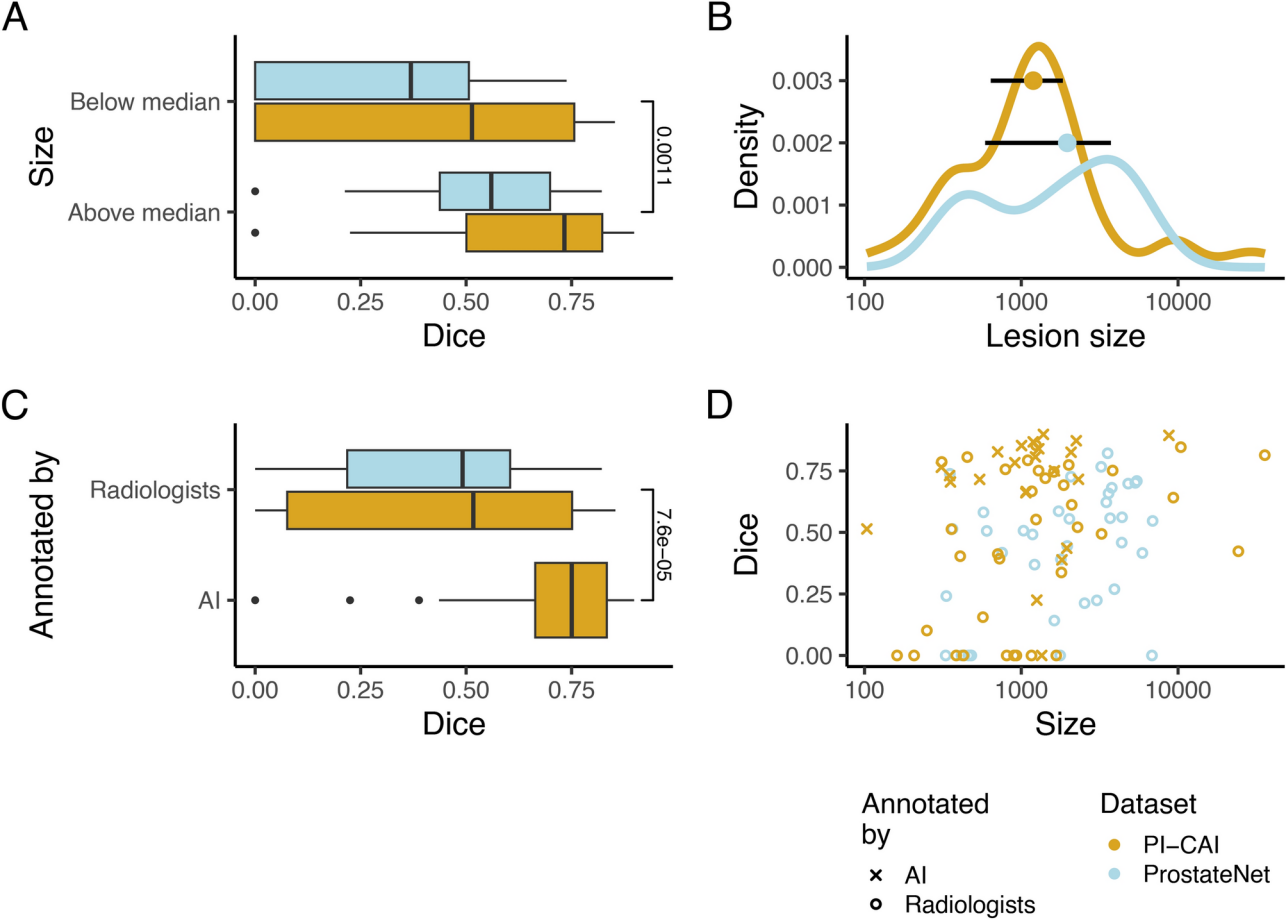
Effect of lesion size and annotation type on performance for the best performing model (bpMRI). (**A**) Performance distribution stratified by dataset and lesion size (below or above median). (**B**) Distribution density for lesion sizes across both datasets. Circles represent the median value while black horizontal lines represent the range between the 1st and 3rd quartiles. (**C**) Performance distribution stratified by dataset and annotation type (whether the lesion was annotated by a radiologist or by an AI model). (**D**) Comparison of lesion size with Dice. Each point corresponds to a case, different shapes correspond to different annotation types. Across all plots, golden and blue correspond to PI-CAI and ProstateNet, respectively. *p*-values in (**A**,**C**) correspond to a two-sided Wilcoxon test.

Additionally, there is a consistently large number of recommended unnecessary biopsies—indeed, for bpMRI PNetCAI models tested on PNet data, 54.05% of cases (n = 120) would have an unnecessary biopsy recommended, while only 17.76% of cases (n = 27) would avoid an unnecessary biopsy. This can have a negative impact on the well-being of individuals who have to undergo these unnecessary biopsies.

**Table 7:**
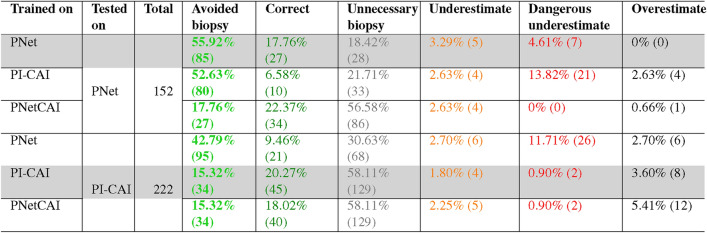
Absolute and relative frequency of bpMRI AI system recommendations, stratified by training and testing dataset. Counts are displayed between brackets after percentages.

### Prospective validation of a simulated clinical decision system

As noted above, an automated system based solely on our models would either lead to dangerous underestimates (i.e. no lesion detected when a lesion was present) or an excess of unnecessary biopsies. To curtail these negative aspects, we devised a clinical decision protocol requiring the interaction of two different decisions, one made by a radiologist (i.e. determine that an individual should have a follow-up biopsy) and the other made by our CAD system: (i) if a radiologist does not recommend a follow-up biopsy, none is performed; (ii) if a radiologist recommends a follow-up biopsy and our model recommends no follow-up biopsy, this is not performed; and (iii) if a radiologist and our model recommend a follow-up biopsy, a biopsy is performed. In effect, this is the ideal case scenario for a model which is highly sensitive but whose specificity is relatively low (i.e. the model produces an excess of false positives).

**Fig. 6:**
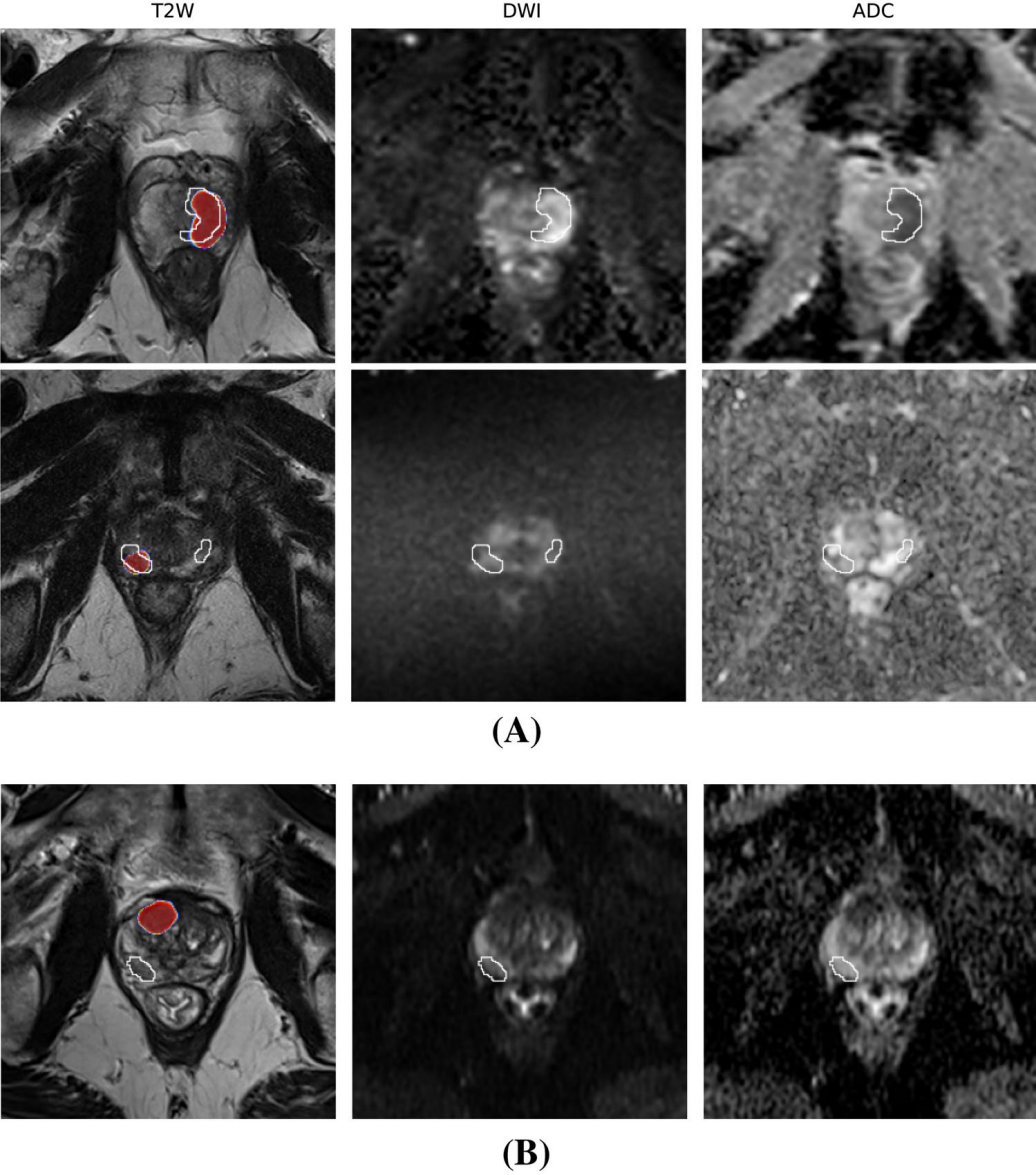
Examples of correctly detected and missed cases. **(A)** Correctly classified and detected lesions. Each row represents a different case selected at random from the correctly detected samples, and the slices shown are those where the index lesion ground truth is most visible in the sequences, **(B)** missed detected example. The slice choice is the same as the one described previously. For both sets of examples, the ground truth is represented by the white outline, allowing for the view of the target region, and the probability maps are only displayed in the T2W images as to not cover the hyper- and hypo- intense areas of both DWI and ADC sequences.

To avoid the self-fulfilling prophecy of developing models and testing them on the same data, we used a ProstateNet prospective cohort of 73 cases (21 aggressive PCa) to determine whether such a strategy could be beneficial. In terms of prospective segmentation and detection performance, these models perform similarly to those trained and tested with retrospective data (Table 8). Lastly, and most importantly, our results show that using a combined CAD system as described above would indeed lead to a reduction of unnecessary biopsies (21.9% of cases [n=16]; Fig. 4) without increasing the dangerous underestimates.

Finally, we assess whether these models are capable of performing reasonably well across different confidence thresholds and whether they can be reliably used at the lesion level. As highlighted in Fig. A.3, these models perform better when confidence thresholds are lower (AUROC is consistently higher when such is the case). Additionally, there is limited applicability for these models as lesion segmentation tools due to their relatively high number of false positives.

**Table 8:** Prospective cohort results. For each model, per sequence, the average Dice, Recall and Precision performances are presented. The best Recall scores are highlighted in bold for easier comparison.

Metric	Modality	PNet	PC	PNetCAI
Recall	T2W	0.71	0.52	0.90
Precision	T2W	0.48	0.25	0.30
# Detected lesions	T2W	15	11	19
Recall	bpMRI	0.86	0.48	**1.00**
Precision	bpMRI	0.54	0.23	0.34
# Detected lesions	bpMRI	18	10	**21**
# Lesions				21

### Determinants of performance

To better understand performance (Dice scores), we analysed distinct factors—lesion size and whether annotations were derived by an AI or by a radiologist. ProstateNet and PI-CAI have different distributions of lesion size (Fig. 5B), with ProstateNet presenting lesions larger than those in PI-CAI. Indeed, at a significance threshold of 0.05, there is a significant Dice difference between below and above median lesions for both datasets (Fig. 5A). While more evident in the ProstateNet dataset, both sets of data exhibit a size bias where larger lesions are easier to segment. Given that some lesions in PI-CAI are generated by an AI model^26^, we compared the Dice scores between lesions annotated by AI and by radiologists, showing that the former lead to higher Dice scores than the latter ($p=7.6e-5$; Fig. 5C). In Fig. 5D, we highlight a more comprehensive vision of these results.

Finally, to acquire a qualitative understanding of prediction quality, we analyzed a subset of true positive and false negative detections at the lesion level for our best-performing model—trained on bpMRI PNetCAI data. Figure 6 offers a concise overview of our analysis, while Figs. A.1 and A.2 present a comprehensive depiction. As highlighted in Fig. A.1, true positives typically encompass all or nearly all of the lesions as annotated by expert radiologists. This is what is expected of such CAD systems, providing information regarding the general area where it thinks the lesion is located to guide the radiologist. When considering negative examples (Fig. A.2), there is a trend—while the lesion annotated by expert radiologists may be missed, the models identify another likely lesion somewhere else in the prostate. In summary, the conclusions derived from our qualitative analysis are as follows:

- In each case, our model detected additional existing lesions and/or cysts. Although these were marked as missed cases due to insufficient overlap with the ground truth mask, they nonetheless correctly identified other lesions as aggressive, demonstrating significant clinical value for a CAD system.
- In some instances (Fig. A.2), with the fourth example being the only visible one in this set of slices, our model correctly identified the area of interest despite low confidence and probability scores. This demonstrates the utility of our model in guiding radiologists to significant areas regardless of the displayed probability.

## Discussion

In this work, we posit a hybrid computer-aided diagnosis (CAD) system combining radiologists and an automatic lesion detection model, which can reduce the number of unnecessary biopsies in the diagnosis of aggressive prostate cancer (ISUP>1) in the general population of patients undergoing biparametric MRI for prostate cancer diagnosis. Through a simulated clinical feasibility scenario, a reduction of approximately 20% of unnecessary biopsies was achieved, with a prospective validation showing that this does not lead to a reduction in the number of detected prostate cancer cases. Ultimately, we highlight how deep-learning methods can assist in the reduction of unnecessary biopsies without leading to decreased sensitivity. This has the potential to reduce patient discomfort and complications following biopsies.

Largely, most CAD systems of the sort seek to solve a similar, albeit separate problem — that of detecting undiagnosed prostate cancer cases with the objective of increasing sensitivity by reducing the amount of false negatives; our approach considers a different problem — that of reducing the number of unnecessary biopsies (i.e. reducing false positives). Indeed, this is also a considerable problem, as a 2019 meta-review showed that the pooled sensitivity for PI-RADS 2.1 was approximately 91% (95% CI=83%-95%)^4^. Works seeking to automate or partially automate prostate cancer diagnosis contemplate strategies focusing either on the detection of lesions with a sufficiently high PI-RADS score (i.e. 3 or 4)^40^ or in the detection of lesions with a confirmed aggressive histological grade (ISUP>1)^25,41,42^. The former has the obvious advantage of requiring no biopsy for training, but hinders the clinical applicability evidenced by the latter. Some of these strategies also incorporate a human-in-the-loop setup, which is more similar to the study design we introduce here^43^. The relevant performance metric which we can compare between our work and previous works is the Recall—we observed a Recall of 82% for models trained/tested on PNetCAI, slightly lower to what has been previously reported (87.2%^43^, 89.4%^25^, 93%^42^). However, we note that these studies are trained/tested on a relatively small number of clinical centers (4 or fewer)^25,42,43^ (which greatly reduces the variability of the data), do not provide confirmation of prospective validation, and do not study the impact of using diverse training datasets on performance. Given the previously reported drop in performance when transferring models between different datasets^10,11,44^ and the fact that models (clinical and otherwise) tend to suffer from temporal degradation^45–47^, such assessments are of paramount importance. Finally, and to the best of our knowledge, our work offers a unique analysis of performance differences when considering lesion size and annotation types, thus better contextualizing results.

This work has some caveats—the simulated clinical scenario does not allow us to estimate the effect of real-world agents (i.e. medical doctors) interacting with such a CAD system. This may lead to optimistic results as automation bias (when users excessively trust the output of automatic CAD systems^48^) can lead to unforeseen outcomes as radiologists may trust excessively in wrong predictions made by CAD systems^49^. It should also be highlighted that, while the best performing model detects all important cases in ProstateNet both retrospectively and prospectively (Figs. 3, 4), not all index lesions are detected, which can cause confusion when results are interpreted in a clinical setting; this is in part largely associated with how these datasets are annotated — indeed, radiologists are tasked with segmenting at least the index lesion, leading to a fair degree of heterogeneity in the annotations. Additionally, performance is relatively poor when we consider the specificity of these models; while this can be improved through the assistance of a radiologist, it should be noted that additional sources of false positive reduction should be taken, such as an auxiliary classification of lesion candidates^50^ or zone-specific PSA density^51^. Furthermore, our approach does not focus on lesion location — particularly, we perform predictions at the patient, rather than at the lesion level — so further studies on this are necessary. Finally, it should be noted that there is no guarantee that nnUNet is the best performing model (“No Free Lunch” theorem) — earlier works have suggested that other models may be better performing than nnUNet for prostate lesion segmentation^50^, so a more comprehensive assessment with other models could be important.

## Supplementary Information

Supplementary Information.

## Data Availability

The datasets generated and/or analysed during the current study are available in the PI-CAI repository, https://zenodo.org/records/6624726. The datasets generated and/or analysed during the current study are not publicly available due to data privacy laws but are available from the corresponding author on reasonable request.
