# Supplementary material for: Effective reduction of unnecessary biopsies through a deep-learning-assisted aggressive prostate cancer detector
Source: Sci Rep. 2025 Apr 30;15:15211. doi: 10.1038/s41598-025-99795-y (PMC12043811; doi:10.1038/s41598-025-99795-y)
Supplement: Supplementary file 1 — Supplementary Information. [file 41598_2025_99795_MOESM1_ESM.pdf]

A Supplementary Material

A.1 Validation metrics

The **Dice Similarity Coefficient** (DSC) is a widely used metric for segmentation tasks, and it measures the spatial overlap between the voxels of the ground truth and predicted masks. The produces score is in the range of $[0,1]$, where one indicates a perfect segmentation.

The **Recall** is the percentage of predictions for which we can count a positive detection of the lesion. A positive detection happens when the predictions has a DS superior to a given threshold, which for the study was defined as 10%. This metric is sometimes also called Sensitivity.

The **Average Symmetric Surface Distance** (ASSD) measures, in millimetres, the difference between the surface voxels of the predicted mask and the ground truth mask. For each surface voxel of both images, the Euclidean distance to the closest surface voxel of the opposite image is calculated using the approximate nearest neighbor technique. All measurements are averaged, with the final score indicating the average distance, where a value of zero indicates a perfect segmentation. In essence, the ASSD provides information about the spatial accuracy of the segmentation (how closely the prediction boundary matches the ground truth boundary).

The **Hausdorff Distance** (HD), also known as Maximum Symmetric Surface Distance, measures, in millimetres, the maximum difference between the surface voxels of the predicted mask and the ground truth mask. The Euclidean distance is measured in the same way as the ASSD, however, the final score will be the maximum distance, or error, between both masks, where a value of zero indicates a perfect segmentation.

The **Relative Absolute Volume Difference** (RAVD) measures, in millimetres, the absolute size difference between the volume of the predicted mask and the ground truth mask. RAVD can be either negative or positive, with negative values denoting smaller and positive values a larger predicted volume compared to the ground truth. Values close to zero indicate both volumes are similar.

A.2 Reducing false positives

In addition to the prostate gland overlap discussed in the main body of the document, we tested several other methods to reduce the number of false positives without increasing the number of false negatives (missed cases). Here, we provide a brief description of the other strategies used that ultimately failed at achieving this objective.

PIRADS

We attempted to use the PIRADS score as a cutoff for the negative cases, classifying all cases with a PIRADS score ≤ 2 as negative. This approach led to a substantial decrease in Recall, increasing the number of false negative predictions, with minimal to none gains in precision. We attribute this to the equivocal nature of the PIRADS 2 and 3 scores, as these were the categories that failed in this approach.

PSA density

We attempted an approach based on the work of Hamm et al.[52], where we calculated the PSA density (PSAd) for each sample, and conducted both a ROC analysis on the training set to define a threshold between clearly negative cases and potentially positive cases, as well as used the thresholds between $[0.1-0.2]$ defined in the literate. While the PI-CAI dataset included the PSAd information for the entire cohort in the metadata, for the ProstateNet data, we only had access to the PSA value. Therefor, used the segmentation model defined by Rodrigues et al.[11] to generate the masks, and use those to calculate the prostate volume. Significant issues emerged due to the substantial discrepancy between ProstateNet and PI-CAI PSAd scores, resulting in distinctly different selected thresholds and underscoring a pronounced domain shift within the data. Subsequently, the application of these thresholds demonstrated a marked decline in Recall, accompanied by only a minimal alteration in precision.

Lesion candidate confidence

Lastly, we conducted a ROC analysis on the confidence threshold for the lesion candidates, aiming to eliminate low-confidence false positives. Similar to other approaches, this resulted in a detrimental effect on the Recall with minimal to no changes in precision.

A.3 Qualitative analysis figures

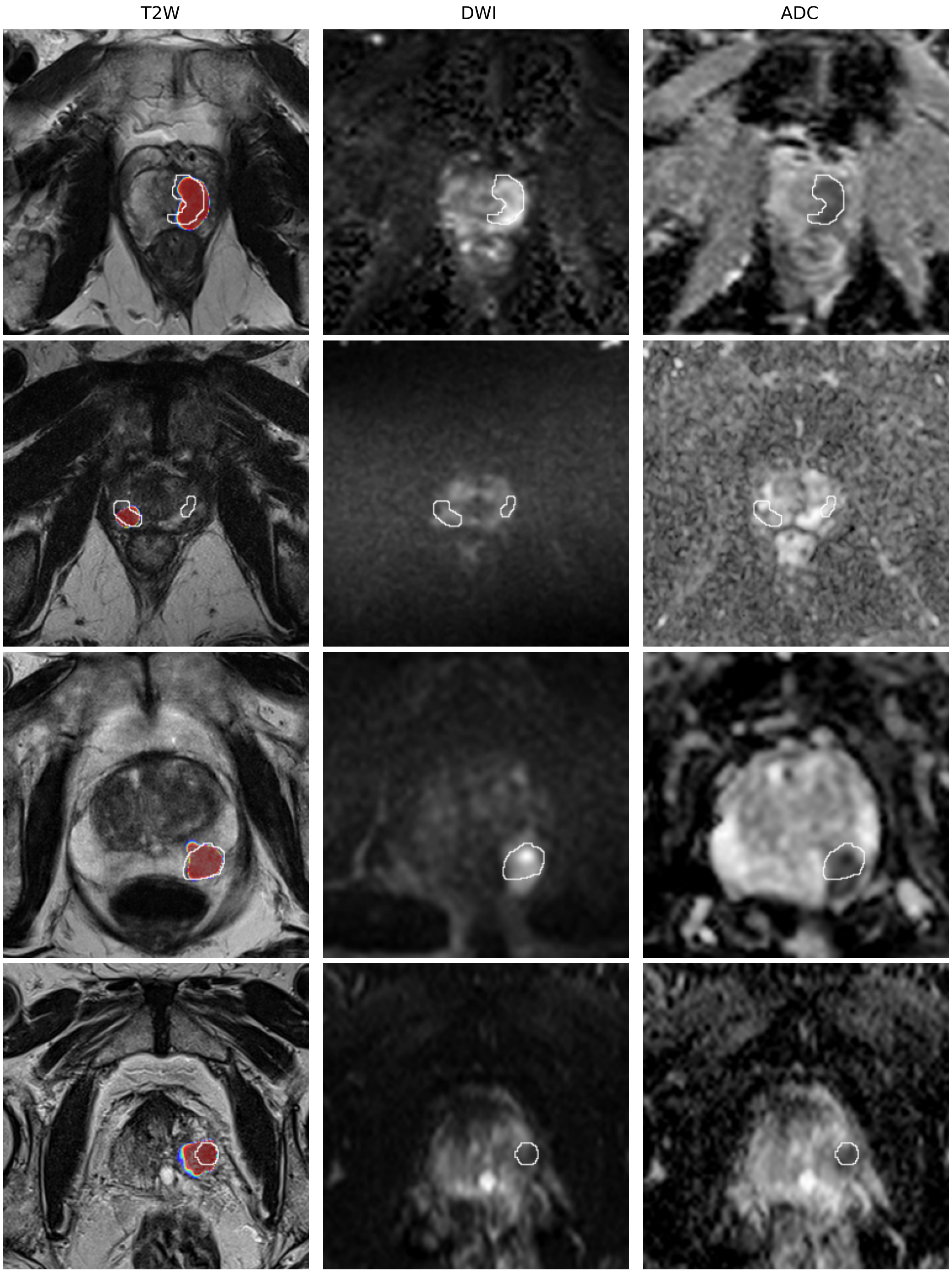

Figure A.1. Correctly detected examples. Instances where our best model (bpMRI PNetCAI) is able to correctly predict there is an aggressive lesion, as well as to correctly locate it in the image. The mask is represented by the white contour in all sequences, while only the T2W image contains the probability map, to facilitate imafe analysis, as both other sequences are more informative.

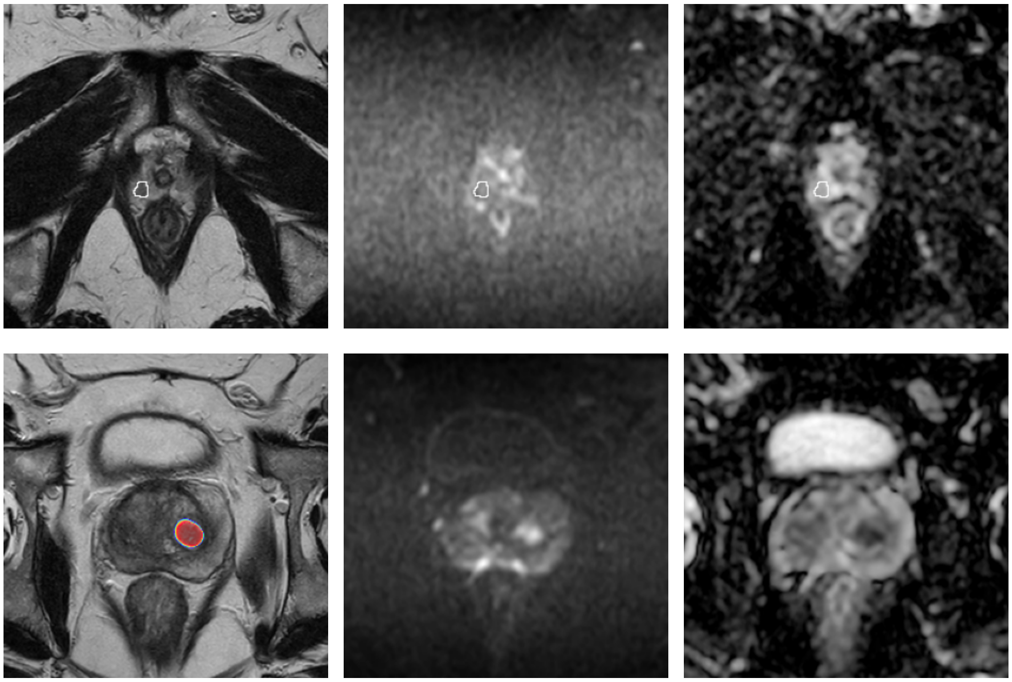

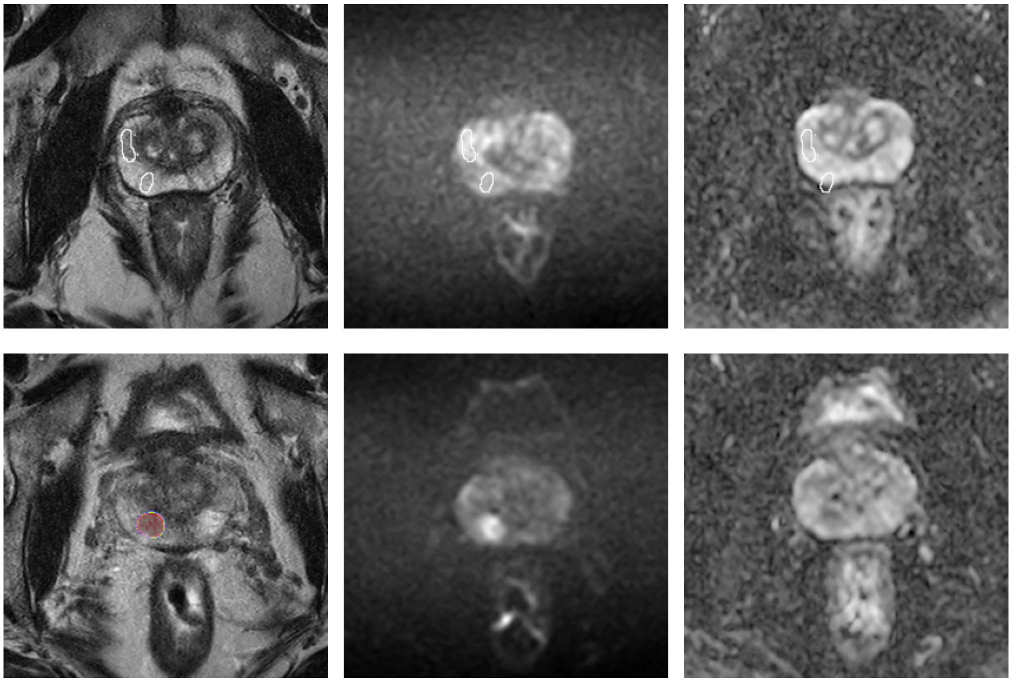

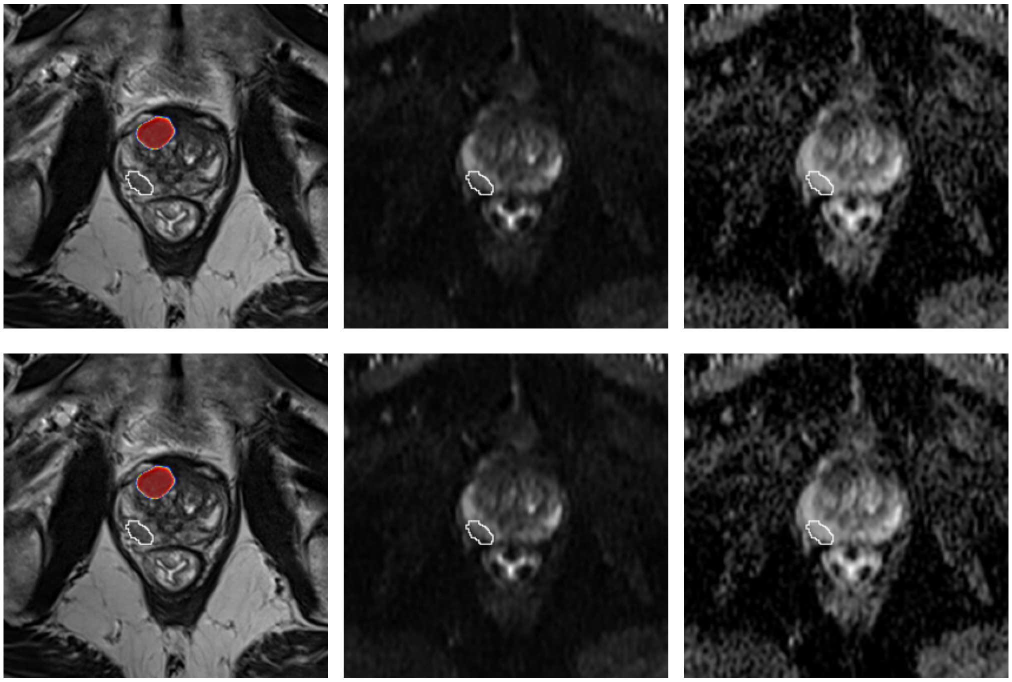

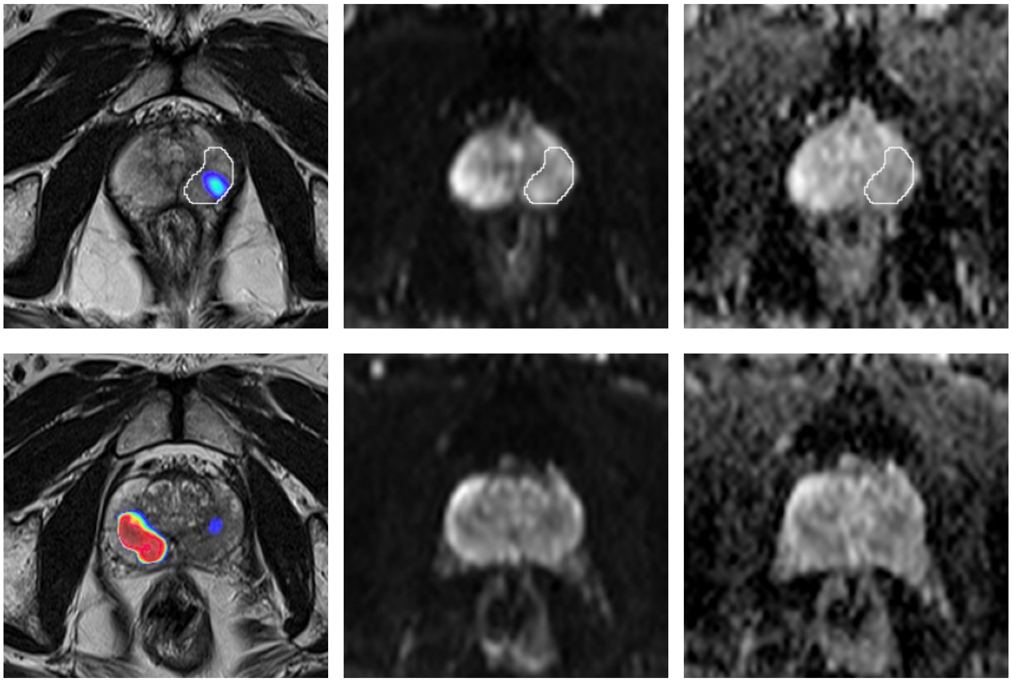

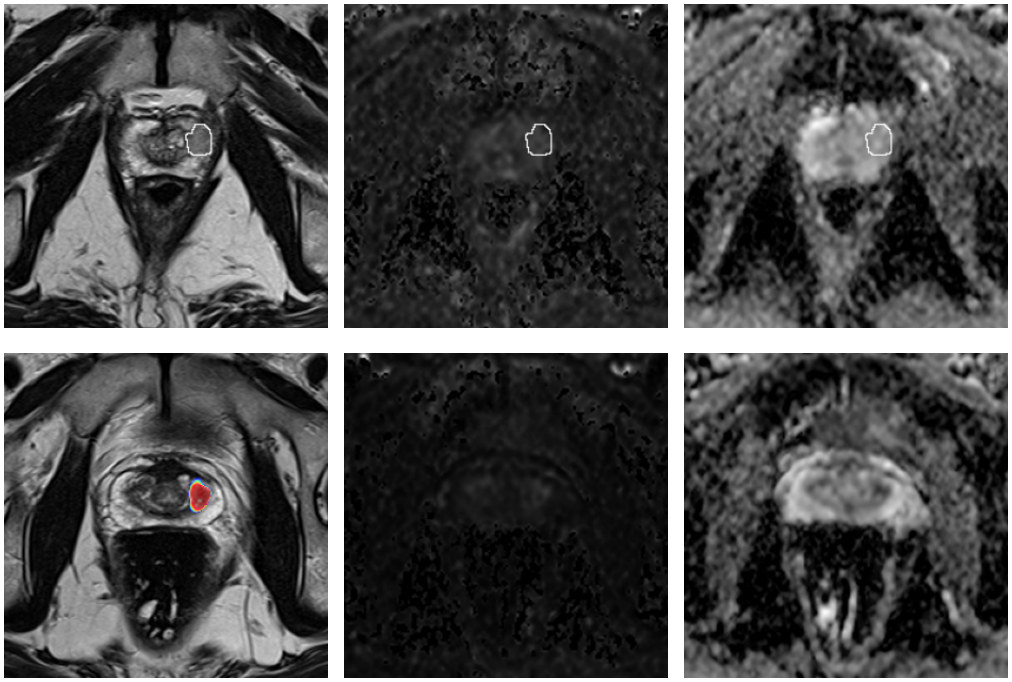

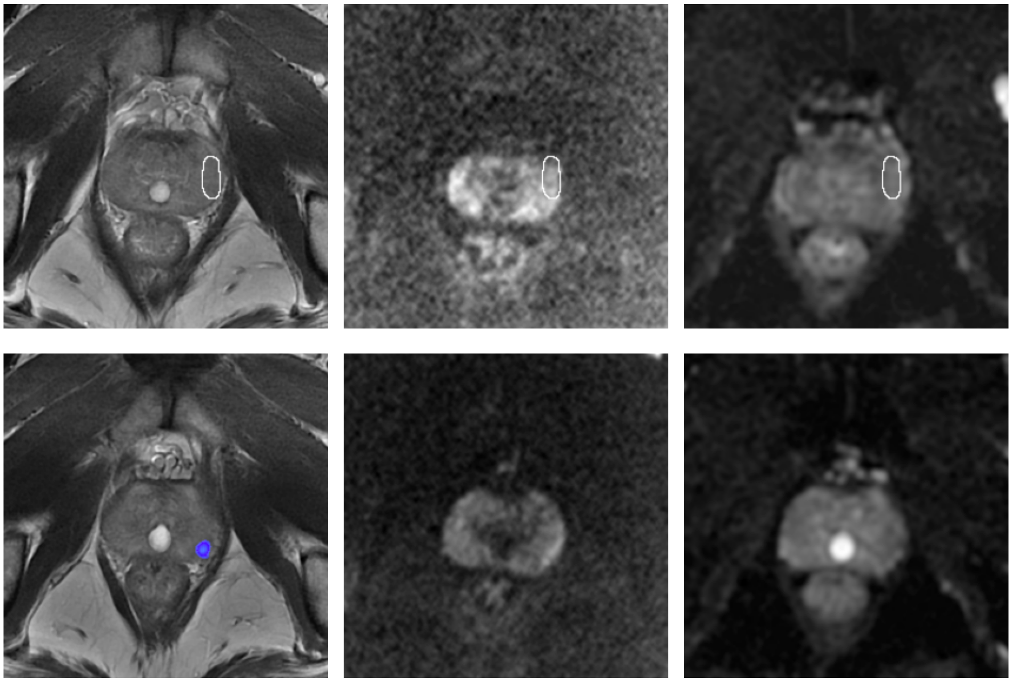

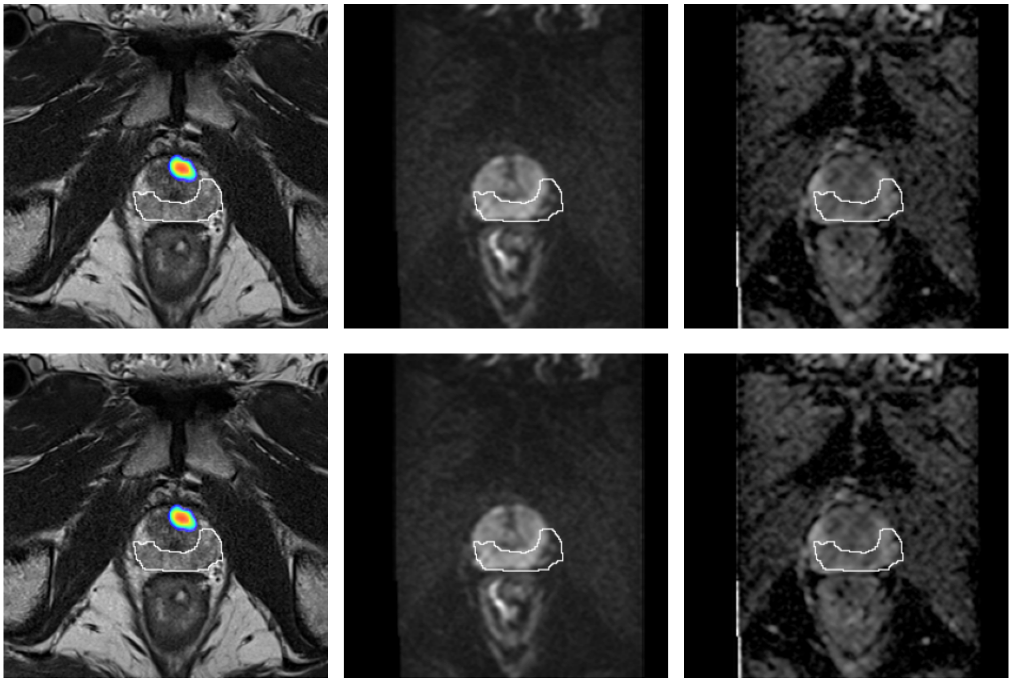

Figure A.2. Wrongly detected cases. Instances where our best model (bpMRI PNetCAI) is able to predict the location of other lesions that are not index lesions, which, despite being correct, are counted as missed cases in our evaluation due to having $\leq$ 10% overlap with the index lesion. The mask is represented by the white contour in all sequences, while only the T2W image includes the probability map to facilitate image analysis, as both other sequences are more informative. Each set of examples includes both the slice where the ground truth index lesion is most visible (ground truth row), as well as the slice where our model exhisbits the highest probability (same slice for examples 3 and 7). Each example is separated by a horizontal divider.

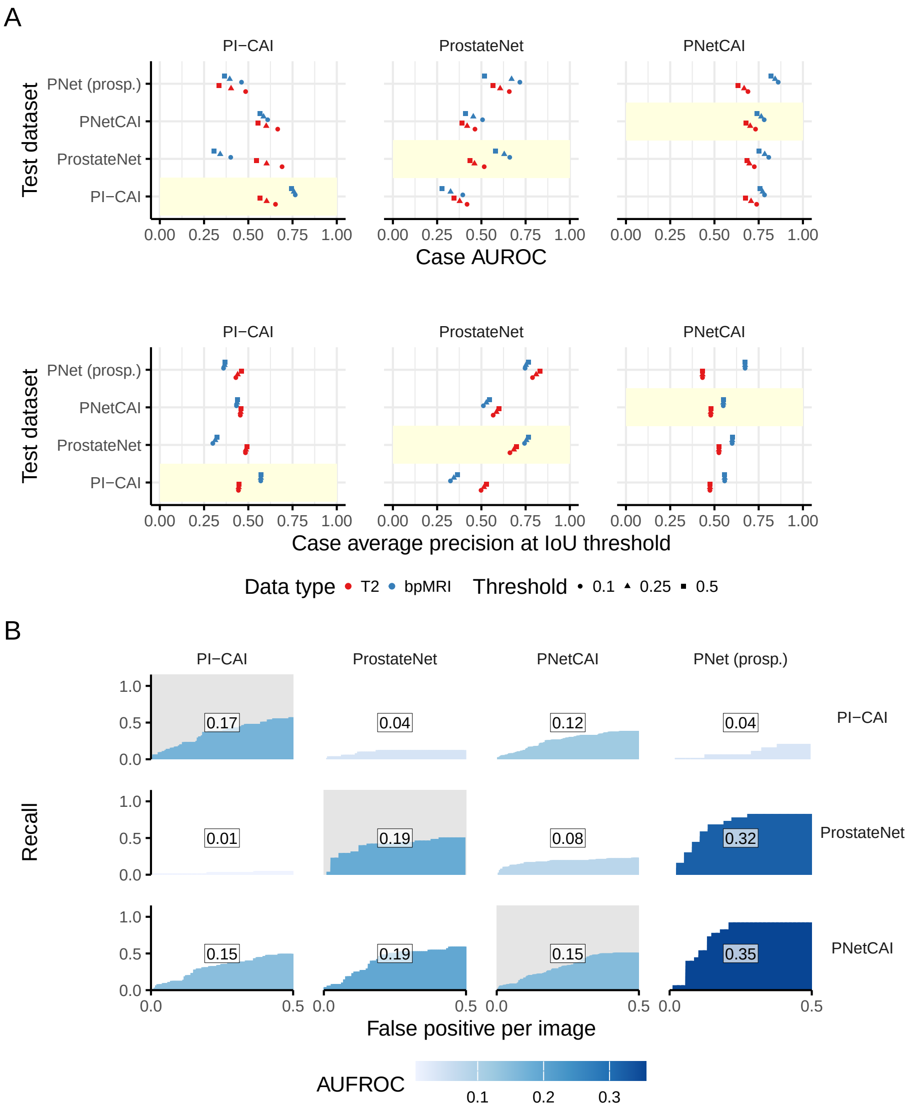

Figure A.3. **Threshold sensitivity analysis and per-lesion metrics. A** — Case area under the receiver operating curve (AUROC) and average precision at 10% intersection over union (IoU) threshold stratified by training and testing data. Colours correspond to the data type (whether T2 or bpMRI were used) and marker shapes represent the threshold. The yellow background represents training and testing on the same dataset. Each column refers to models trained on a specific dataset (i.e. PI-CAI, ProstateNet (or PNet) and PNetCAI). **B** — Free response operator curve (FROC) stratified by training and testing data for biparametric MRI models. Colours correspond to the area under the partial FROC curve. Grey background represents training and testing on the same dataset. Panel rows correspond to models trained on PI-CAI, ProsatateNet or PNetCAI training data, respectively, whereas panel columns correspond to models tested on PI-CAI, ProstateNet, PNetCAI and PNet (prosp.) hold-out test sets, respectively. For both **A** and **B**, "PNet (prosp.)" refers to prospective ProstateNet data.
